# Protecting Mammalian Hair Cells from Aminoglycoside-Toxicity: Assessing Phenoxybenzamine’s Potential

**DOI:** 10.3389/fncel.2017.00094

**Published:** 2017-04-18

**Authors:** Paromita Majumder, Paulette A. Moore, Guy P. Richardson, Jonathan E. Gale

**Affiliations:** ^1^UCL Ear Institute, University College LondonLondon, UK; ^2^Sussex Neuroscience, School of Life Sciences, University of SussexFalmer, UK; ^3^Department of Cell and Developmental Biology, University College LondonLondon, UK

**Keywords:** hair cells, cochlea, aminoglycosides, ototoxicity, FM 1-43, organotypic culture, mechanoelectrical transduction channels, inner ear

## Abstract

Aminoglycosides (AGs) are widely used antibiotics because of their low cost and high efficacy against gram-negative bacterial infection. However, AGs are ototoxic, causing the death of sensory hair cells in the inner ear. Strategies aimed at developing or discovering agents that protect against aminoglycoside ototoxicity have focused on inhibiting apoptosis or more recently, on preventing antibiotic uptake by the hair cells. Recent screens for ototoprotective compounds using the larval zebrafish lateral line identified phenoxybenzamine as a potential protectant for aminoglycoside-induced hair cell death. Here we used live imaging of FM1-43 uptake as a proxy for aminoglycoside entry, combined with hair-cell death assays to evaluate whether phenoxybenzamine can protect mammalian cochlear hair cells from the deleterious effects of the aminoglycoside antibiotic neomycin. We show that phenoxybenzamine can block FM1-43 entry into mammalian hair cells in a reversible and dose-dependent manner, but pre-incubation is required for maximal inhibition of entry. We observed differential effects of phenoxybenzamine on FM1-43 uptake in the two different types of cochlear hair cell in mammals, the outer hair cells (OHCs) and inner hair cells (IHCs). The requirement for pre-incubation and reversibility suggests an intracellular rather than an extracellular site of action for phenoxybenzamine. We also tested the efficacy of phenoxybenzamine as an otoprotective agent. In mouse cochlear explants the hair cell death resulting from 24 h exposure to neomycin was steeply dose-dependent, with 50% cell death occurring at ~230 μM for both IHC and OHC. We used 250 μM neomycin in subsequent hair-cell death assays. At 100 μM with 1 h pre-incubation, phenoxybenzamine conferred significant protection to both IHCs and OHCs, however at higher concentrations phenoxybenzamine itself showed clear signs of ototoxicity and an additive toxic effect when combined with neomycin. These data do not support the use of phenoxybenzamine as a therapeutic agent in mammalian inner ear. Our findings do share parallels with the observations from the zebrafish lateral line model but they also highlight the necessity for validation in the mammalian system and the potential for differential effects on sensory hair cells from different species, in different systems and even between cells in the same organ.

## Introduction

Aminoglycosides (AGs) are widely used antibiotics because of their low cost and high efficacy against gram-negative bacterial infection. AGs are both nephrotoxic (Toubeau et al., 1986; Hock and Anderson, 1995) and ototoxic (Greenwood, 1959) affecting the vestibular and auditory organs (Matz et al., 2004). Among the AGs neomycin is highly toxic, particularly in the hearing organ, the cochlea (Forge and Schacht, 2000). In the cochlea, AGs first affect the high-frequency hair cells found at the basal end of the cochlea, subsequently extending to the lower-frequency regions at the apical end of cochlear spiral (Aran and Darrouzet, 1975; Stebbins et al., 1979; for review see Forge and Schacht, 2000).

In order to access the apical surface of hair cells AGs must first pass through the blood labyrinth barrier which is composed of a highly specialized capillary network in the stria vascularis (Shi, 2016). Studies with fluorescently-tagged gentamicin showed that it travels from the stria vascularis capillaries into marginal cells and then into the endolymph (Wang and Steyger, 2009; Wang et al., 2010) at which point it comes into contact with the apical surface of the hair cells. AGs can enter into hair cells via an endocytic route (Hashino and Shero, 1995) or via the mechanotransduction (MET) channels, located in the hair cell’s stereociliary bundle (Gale et al., 2001; Marcotti et al., 2005; Dai et al., 2006; Wang and Steyger, 2009; Alharazneh et al., 2011) a route that is facilitated by the low calcium concentration of the endolymph (Marcotti et al., 2005).

When mammalian cochlear hair cells die there is no endogenous regenerative process that can replace them and thus hearing loss is permanent. Stem cell based therapies are being pursued and may well be available in the longer term. In the shorter term, however, it is important that we discover therapies that might protect hair cells from damage and death as a result of ototoxic medication, noise-induced hearing loss and aging.

New aminoglycoside antibiotics derived from the structural backbones of existing AGs are being produced to generate less ototoxicity whilst still being effective antibiotics (Matt et al., 2012; Duscha et al., 2014; Shulman et al., 2014; Huth et al., 2015). Until such antibiotics are available, a protective agent that could be applied along with any known ototoxin would be very advantageous. There have been a number of screens to identify compounds that might confer hair-cell protection during ototoxicity, including screens of FDA-approved drugs that could bypass some of the approval requirements (Ou et al., 2009). Many of these have used the zebrafish lateral line system as a screening model. One of the compounds that came through two separate screens using two different sets of FDA-approved compounds was phenoxybenzamine (also known as N-(2-chloroethyl)-N-(1-methyl-2-phenoxyethyl) benzylamine hydrochloride or dibenzyline) which was one of seven drugs which showed protection against aminoglycoside-induced hair-cell death potentially by blocking aminoglycoside uptake by hair cells (Ou et al., 2009; Vlasits et al., 2012). In zebrafish it was noted that pre-incubation of phenoxybenzamine was required to see efficacy in increasing hair-cell survival (Vlasits et al., 2012). Although phenoxybenzamine is a well characterized drug that is known to cross the blood brain barrier (Diop and Dausse, 1986) and so perhaps the blood labyrinth barrier, it has not been tested on mammalian cochlear hair cells.

Phenoxybenzamine is a well-known alpha-1-A and alpha-2-A adrenergic receptor antagonist (Thoenen et al., 1964). Among alpha 1-A and 2-A receptors, phenoxybenzamine has a slow onset and long lasting effect compared with other alpha adrenergic blockers and it is used to reduce the vasoconstriction caused by epinephrine (adrenaline) and norepinephrine. Phenoxybenzamine acts by covalently binding to the receptor resulting in the inhibition of ligand binding. It is used to reduce the hypertension caused by pheochromocytoma, for lower urinary tract problems (e.g., neurogenic bladder; Te, 2002), benign prostatic hyperplasia (Kumar and Dewan, 2000) and in complex regional pain syndrome (Inchiosa, 2013). Phenoxybenzamine is also known to block 5-HT2 serotonin receptors (Frazer and Hensley, 1999), in some cases acting as an irreversible blocker (Doggrell, 1995).

In this study we evaluated whether the protective effects of phenoxybenzamine that are observed in zebrafish hair cells (Ou et al., 2009; Vlasits et al., 2012) are also observed in mammalian inner hair cells (IHCs) and outer hair cells (OHCs). We first investigated the effects of phenoxybenzamine on the rapid FM1-43 entry into hair cells that occurs via the mechanoelectrical-transduction channels and mimics aminoglycoside entry (Gale et al., 2001). Second, we tested whether phenoxybenzamine could protect hair cells during neomycin ototoxicity.

## Materials and Methods

### Materials

Phenoxybenzamine hydrochloride was obtained from Santa Cruz Biotechnology, Dallas, TX, USA and neomycin and ciprofloxacin from Sigma-Aldrich, UK. Leibovitz’s L15 (L15), DMEM/F12, FM1-43FX and fetal bovine serum (FBS) were acquired from ThermoFisher scientific, UK and the cell and tissue adhesive Cell-Tak was supplied by BD Biosciences, UK. All other chemicals were obtained from Sigma-Aldrich, UK.

### Postnatal Cochlea Isolation and Culture

In accordance with the United Kingdom Animals (Scientific Procedures) Act of 1986 (Schedule 1 procedures), cochleae were extracted from C57BL/6 wild-type mice at postnatal day 3–6 (P3-P6). The isolated cochleae were micro-dissected in Leibovitz L15 medium. The apical cochlear turn was discarded and the remaining 1.5 turns separated into “middle” and “basal” coils. The stria-vascularis was kept in place and Reissner’s membrane was cut allowing the cochlear coils to be “fileted” open and then placed onto Cell-Tak® coated Matek (Ashland, MA, USA) dishes (Majumder et al., 2015). Explant cultures were incubated in DMEM/F12 supplemented with 1% FBS and ciprofloxacin (10 μg/ml) at 37°C in a 5% CO_2_/95% air atmosphere overnight prior to experimentation.

### Live FM1-43 Imaging and Analysis

All experiments were conducted at room temperature (20–22°C). FM1-43 (6 μM) was prepared from a 10 mM stock solution. The control culture was loaded with 6 μM FM1-43 for 30 s and then immediately washed (five 10 s washes, followed by three slower, 2 min washes) in L15 or HBHBSS prior to imaging with a Zeiss 510NLO upright confocal microscope (excitation 488 nm, emission 500–550 nm) using a 40× (NA 0.8) water immersion objective. Confocal image stacks (~20 planes, 2 μm intervals) were acquired exactly 20 min after exposure to FM1-43. The confocal laser power and gain settings were identical for all live imaging experiments to enable data comparison. To evaluate whether phenoxybenzamine was more effective when pre-incubated, in a subset of experiments it was applied for 30 or 60 min prior to FM1-43 loading and the loading was performed in the continued presence of phenoxybenzamine (including the washout solution). To test for reversibility of the block, after 30 min of phenoxybenzamine incubation explants were washed for varying amounts of time before testing with FM1-43. Images were analyzed using ImageJ (NIH, USA). A running two frame average was applied to the *Z* planes and to maintain consistency ROIs were drawn two planes (i.e., ~4 μm) below the FM1-43 fluorescence signal from the hair-cell stereocilia. ROIs covered the extent of the cell body in that plane. Average intensities from the ROIs were recorded and the background fluorescence (measured in a non-cellular region) was subtracted. Measurements were taken from 30 OHCs and 10 IHCs per explant.

### Ototoxic Hair Cell Death and Protection Assay

To determine a dose-response curve, middle and basal coil cochlear explants were exposed to 0, 10, 100, 200, 250, 400 or 1000 μM neomycin for 24 h. To determine whether phenoxybenzamine confers protection against neomycin ototoxicity, cochlear explants were pre-treated for 1 h in 0, 50, 100 or 200 μM phenoxybenzamine followed by 24 h co-treatment in phenoxybenzamine and 250 μM neomycin in DMEM/F12 media at 37°C in a 5% CO_2_/95% air atmosphere. After all experiments, explants were fixed with 4% paraformaldehyde in 0.1 M phosphate buffered saline (PBS, pH 7.2) at room temperature for 30–45 min for immunostaining and later evaluation for pyknotic and surviving hair cells.

### Immunohistochemistry, Image Acquisition and Analysis

After fixation the explants were rinsed three times with PBS and incubated in blocking solution (PBS, 10% secondary host antibody serum and 0.5% Triton X-100) for 2 h. Subsequently, the explants were incubated with a mouse monoclonal anti-myosin 7A antibody, deposited to the DSHB by Orten, D.J. (DSHB Hybridoma Product MYO7A 138-1, used at 1:250) or a rabbit polyclonal anti-myosin 7A (25–6790, Proteus BioScience, used at 1:1000) primary antibody in blocking solution overnight at 4°C. Samples were then washed in PBS and incubated for 2 h at room temperature with 4′,6′-diamidino-2-phenylindole (DAPI 1 μM), AlexaFluor647 phalloidin (33 nM) and goat anti-rabbit-Atto488 or goat anti-mouse-Atto488 secondary antibodies in blocking solution. The explants were rinsed three times with PBS and imaged using the multiphoton Zeiss 510 NLO upright confocal microscope. DAPI was imaged using the a two-photon Chameleon-XR Ti:Sapphire laser tuned to 720 nm (435–485 nm bandpass filter), Atto488 was imaged using the 488 nm (500–550 nm bandpass filter) and AlexaFluor647 phalloidin using the 633 nm (long pass filter 650 nm) laser lines. Images were acquired at 1.5 μm *Z* intervals using either Achroplan 40× (NA 0.8) or Achroplan 63× Vis-IR (NA 1.0) water immersion objectives.

**Figure 1:**
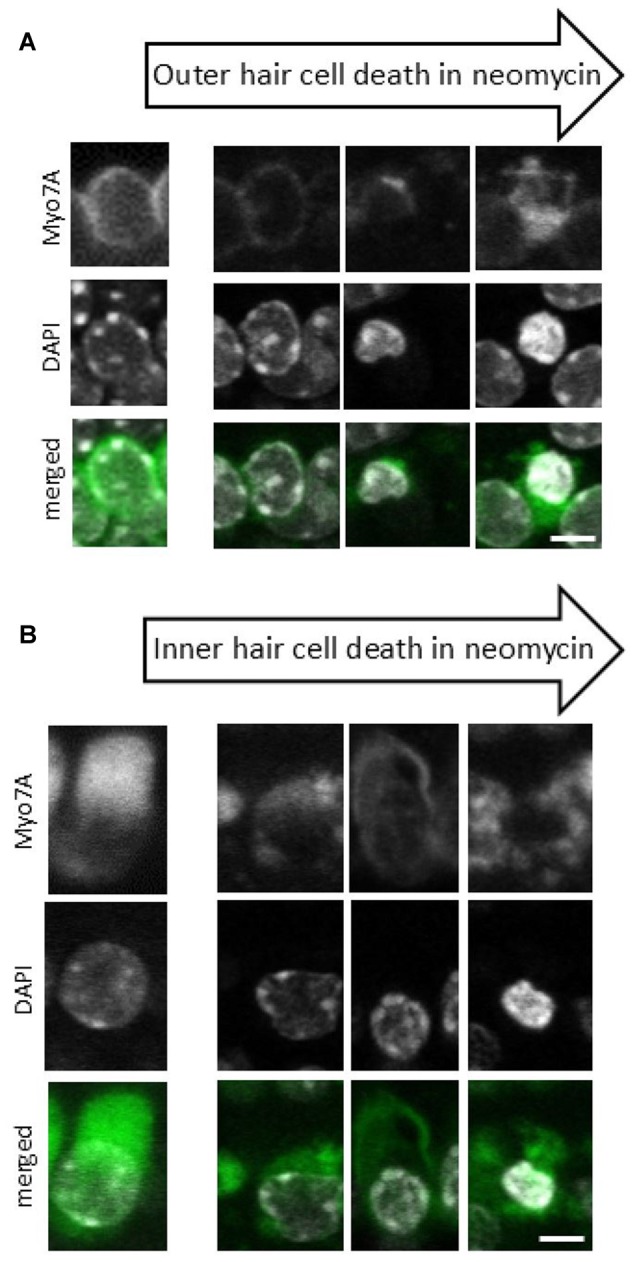
**Progression of neomycin-induced hair cell death.** A sequence of images representing different stages of cell death that are observed after 24 h of neomycin exposure for both outer hair cells (OHCs) **(A)** and inner hair cells (IHCs) **(B)**. Anti-myosin 7A labeling (upper panel), 4′,6′-diamidino-2-phenylindole (DAPI) staining of nuclear chromatin (middle panel), Myo7A/DAPI merged image (lower panel). Since individual cells undergo cell death at different times each cell is at a different stage in the cell death process and the images have been compiled into a pseudo-time series as indicated by the arrows. Scale bar 5 μm.

*Z* stacks (25–30 planes, 1.5 μm intervals) were acquired from two different regions in each explant. Image stacks were visualized using ZEN Lite software (Carl Zeiss Ltd, Germany) to identify pyknotic and surviving hair cells. The hair cells were considered pyknotic when they presented with condensed and marginated chromatin in the nuclei (Li et al., 1995; Lahne and Gale, 2008). Here we have used the characteristic pattern of pyknosis that we observe with the DAPI and myosin 7A staining during the death of IHCs and OHCs (Figure 1).

Hair cells with normal nuclei were considered to be viable surviving hair cells. Counts from the two regions sampled were averaged so that each explant counted as one N sample. Images were independently evaluated by two individuals.

### Statistics

For live imaging experiments, all the data are presented as the mean ± SEM and ANOVA was applied with *post hoc* Tukey-Kramer’s with significance set at *p* < 0.05. For hair-cell survival and pyknosis, all the data are presented as the mean ± SEM and the statistical test used was Students’s *t*-test with significance set as *p* < 0.05.

## Results

### Phenoxybenzamine Blocks Rapid FM 1-43 Entry in Mammalian Hair Cells if Preincubated

**Figure 2:**
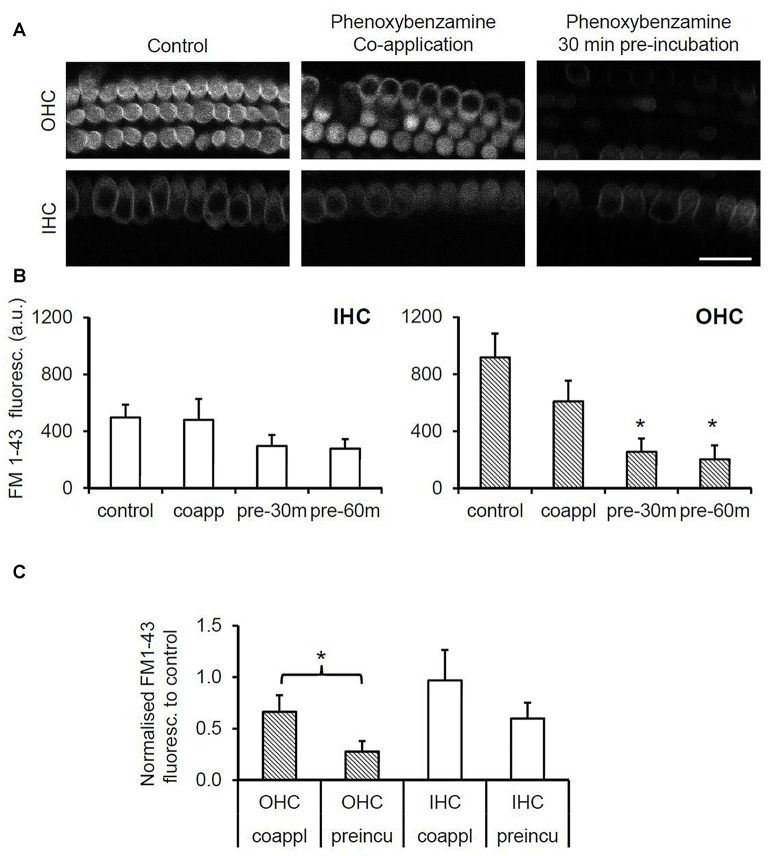
**Phenoxybenzamine (PB) significantly reduces rapid FM1-43 uptake in OHCs but not in inner cells. (A)** Confocal images of FM1-43 uptake after brief exposure (30 s) in OHCs (upper panels) and IHCs (lower panel) under control conditions (left panel), either when co-applied with 100 μM PB (middle panel) or when explants were pre-incubated in PB for 30 min prior to FM1-43 application (right panel). **(B)** Quantification of FM1-43 fluorescence reveals that pre-incubation with phenoxybenzamine for 30 min or 60 min significantly reduces uptake in OHCs but not IHCs. Maximal inhibition of FM1-43 uptake is achieved in 30 min. **(C)** Pre-incubating with phenoxybenzamine is significantly more effective at blocking the rapid uptake of FM1-43. Expressed as a % of control, the uptake was reduced from 66% to 28% in OHCs and from 97% to 60% in IHCs. N numbers: control = 12; 100 μM PB-co-app = 6; 30 min pre-incubation = 9; 60 min pre-incubation = 6. Statistical test: ANOVA Tukey-Kramer’s test, *p < 0.05 in **(B)**; Student’s *t*-test, *p < 0.05 in **(C)**.

**Figure 3:**
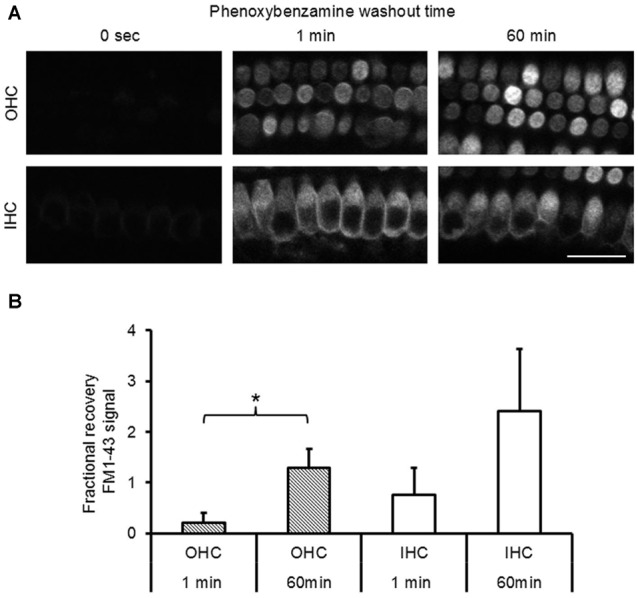
**Phenoxybenzamine block of FM1-43 entry is reversible. (A)** After a 30 min pre-incubation in 100 μM phenoxybenzamine explants were washed for 1 min or 60 min in HBSS before being exposed to 6 μM FM1-43 for 30 s. Confocal images of the FM1-43 fluorescence signal in OHCs (upper panels) and IHCs (lower panel) when applied at 0 s, 1 min or 60 min after phenoxybenzamine washout. **(B)** The block by phenoxybenzamine is completely reversed 60 min after wash out. The fractional recovery of the FM1-43 signals was calculated from *F*_recov_ = (*F* − *F*_min_)/(*F*_max_ − *F*_min_), where *F*_min_ is the signal from 30 min pre-incubation (*n* = 9) and *F*_max_ = control (no phenoxybenzamine) signal (*n* = 12) and *F* = 1 min (4) or 60 min (6). A *F*_recov_ value of 1 is equal to control (*F*_max_). Student’s *t*-test; *p < 0.05.

To evaluate the nature of phenoxybenzamine’s interaction with mammalian MET channels, we measured FM1-43 uptake by hair cells in cochlear explant cultures from postnatal day 3–6 mice. A brief (30 s) exposure to FM1-43 ensured that rapid entry through the MET channels was the primary uptake pathway as opposed to a slower endocytic mechanism. FM1-43 uptake was hair cell specific and irreversible with OHCs taking up approximately twice as much FM1-43 as IHCs as described previously (Gale et al., 2001). When 100 μM phenoxybenzamine was simply co-applied during FM-143 exposure there was no significant effect on FM1-43 uptake in either IHCs or OHCs. In contrast, when phenoxybenzamine was also pre-incubated for either 30 or 60 min, we found a significant reduction in FM1-43 uptake in OHCs, and a small reduction in uptake in IHCs that failed to reach significance (Figures 2A,B). Pre-incubation substantially reduced FM1-43 uptake to 28% of control levels in OHCs and 60% in IHCs respectively, significantly different to uptake both in the controls and in samples in which compounds were co-applied together (Figure 2C). The block of FM1-43 uptake was reversible upon washout although the recovery from block was not immediate. Comparing a 1 min washout with a 60 min washout we can see that the recovery was ~15% in OHCs after 1 min but was complete after 60 min (Figure 3). Recovery appeared to be faster but more variable in IHCs and on average we observed an overshoot in the recovery, but we note that the blocking effect of phenoxybenzamine was less robust in IHCs than in OHCs.

**Figure 4:**
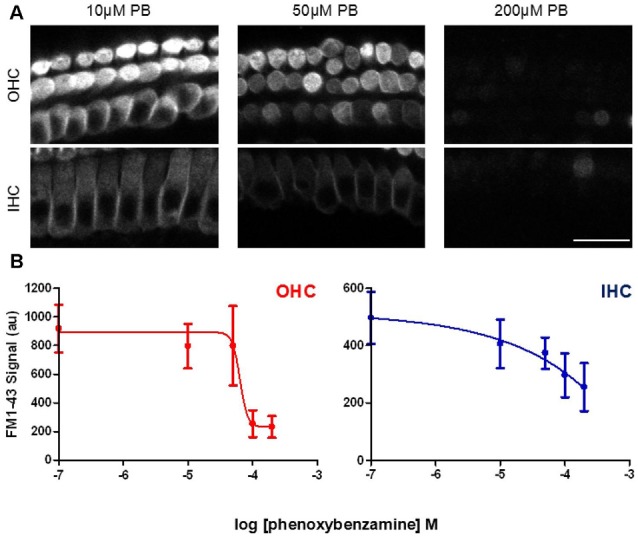
**Phenoxybenzamine block of FM1-43 entry is dose-dependent. (A)** Confocal images of rapid FM-143 uptake assessed after 30 min of pre-incubation in 10, 50 or 200 μM phenoxybenzamine. Images at the focal planes of the OHC and IHC are shown. **(B)** Quantification of the rapid FM1-43 uptake in the presence of different concentrations of phenoxybenzamine reveal the reduction in FM1-43 uptake as a function of increasing phenoxybenzamine concentration in both OHC (red) and IHC (blue). The IC50 and Hill coefficient values from the curve fits are provided in the main text. N numbers: control (12); 10 μM PB (3); 50 μM PB (4); 100 μM (9); 200 μM (4). Scale bar: 20 μm.

In order to determine whether the blockade of FM1-43 entry was dose-dependent, we varied the concentrations of phenoxybenzamine from 0 μM to 200 μM, pre-incubating for 30 min prior to FM1-43 application. The IC50 for the block of FM1-43 uptake by phenoxybenzamine differed slightly between OHC (logIC50 −4.185, 65 μM) and IHC (logIC50 −4.424, 38 μM) however, the Hill coefficient was strikingly different being 4 for OHC and 0.9 for IHC (Figure 4). One simple interpretation of these data is that there is a difference in the molecular nature of the MET channel in the two cochlear hair cell types. An alternative explanation is that the MET channel activity is reduced indirectly via a process that is affected by phenoxybenzamine and that is more prevalent in OHCs. We note that higher concentrations of phenoxybenzamine could not be tested due to problems with solubility that have been reported by others (Nisa et al., 2010).

### Phenoxybenzamine Can Protect Hair Cells from Neomycin-Induced Ototoxicity

**Figure 5:**
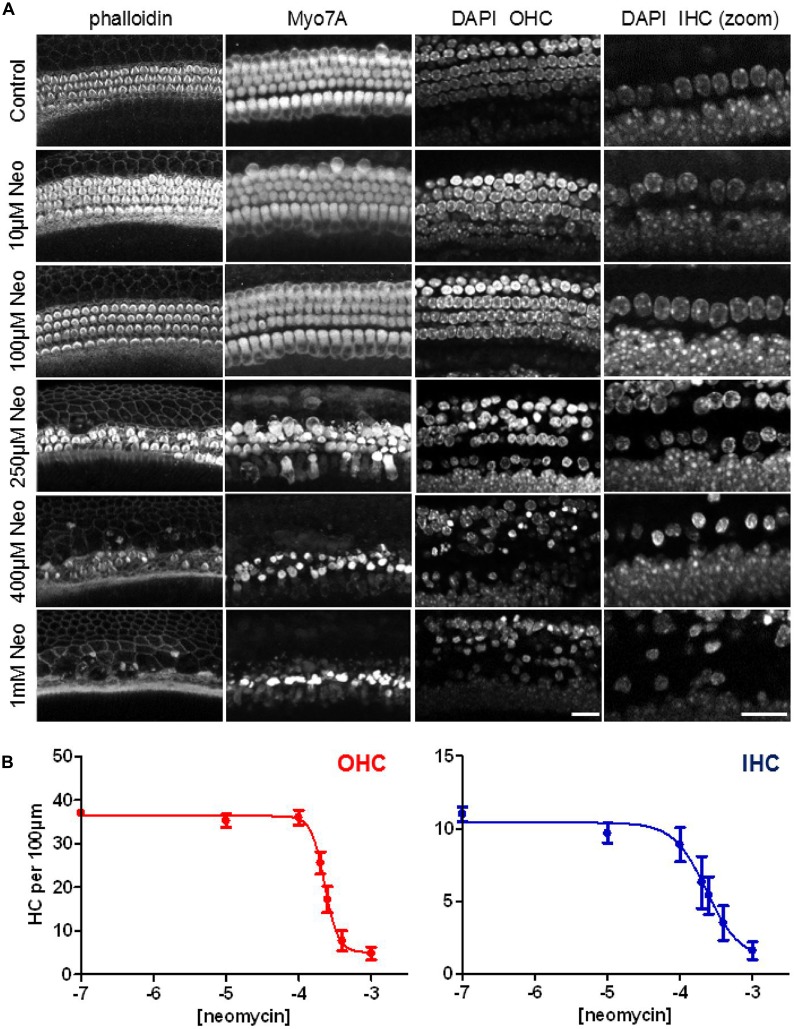
**Steep dose-dependent increase in hair-cell death after 24 h exposure to neomycin. (A)** Confocal images of three different cellular markers: phalloidin, labeling F-actin; myosin7A (Myo7A), for the hair cell soma and DAPI which showing nuclear condensation and pyknosis in neomycin-treated examples. The right hand panels show a higher magnification (zoom) of the DAPI label in the IHC region. **(B)** Quantification of the numbers of surviving hair cells (expressed per 100 μm) reveals the steep dose-dependency of neomycin toxicity after 24 h of drug exposure. The 50% cell-death constants are similar between OHC (228 μM) and IHC (232 μM) but the Hill coefficients were quite different (4.7 and 1.9 respectively). N numbers: 0.1 μM (10); 10 μM (9); 100 μM (9); 200 μM (5); 250 μM (9); 400 μM (8); 1000 μM (10). Scale bar: 20 μm.

For the next experiments we set out to determine a concentration of neomycin that gave us ≥50% toxicity in our mouse cochlear explant culture model after 24 h of exposure. Cochlear explants were treated with neomycin at a range of concentrations from 0 μM to 1000 μM. At the end of the 24-h exposure explants were fixed and triple-labeled with DAPI, fluorescent phalloidin and antibodies to myosin 7A. *Z*-stack images were then collected using a confocal microscope (Figure 5A). Since hair cells are known to be able to survive without a hair bundle (Gale et al., 2002), we focused our analysis on the hair-cell soma and nuclear chromatin markers, as shown in Figure 1, to quantify the number of surviving hair cells, using the image stacks. A steep dose-dependence was observed for neomycin-induced hair cell death for both OHC and IHC (Figure 5B), with 50% cell death constants of 228 μM (log −3.642) and 232 μM (log −3.635) and different Hill coefficients of 4.7 and 1.9, respectively. Based on these experiments we decided to use 250 μM neomycin in subsequent experiments to test whether phenoxybenzamine could provide any protection in this 24 h ototoxicity model.

**Figure 6:**
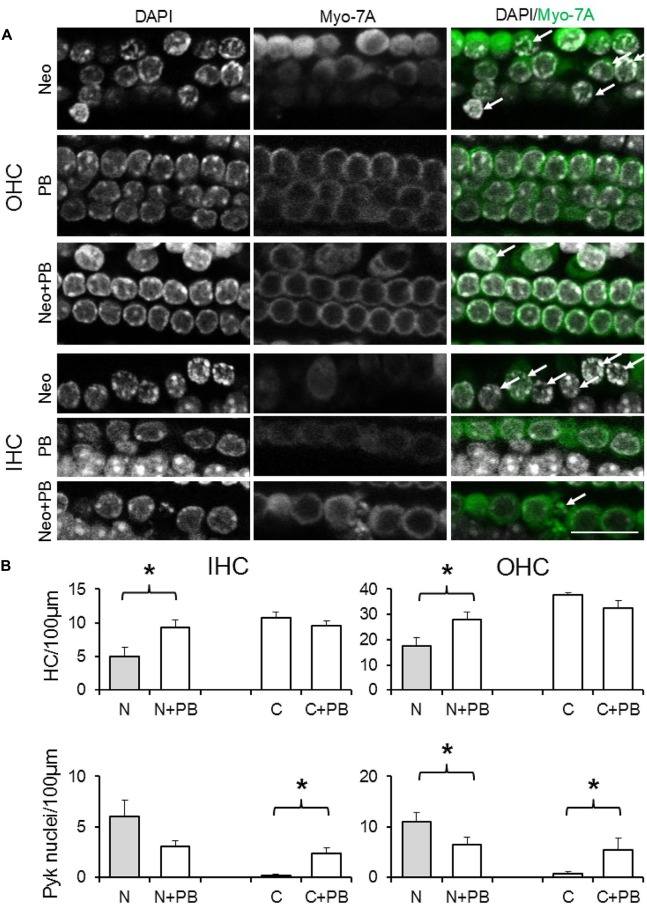
**Phenoxybenzamine confers protection against neomycin ototoxicity. (A)** Representative confocal images showing the protective effect of the 100 μM phenoxybenzamine on the neomycin-induced hair cell death in both OHC and IHC. Images for phenoxybenzamine alone (PB), neomycin-alone for 250 μM for 24 h (Neo) or pre-incubation and continued presence of phenoxybenzamine during the neomycin exposure (Neo+PB). Images showing condensed chromation and pyknotic nuclei (DAPI staining, arrows) and the absence of the myosin7A labeling in missing/damaged hair cells. **(B)** Quantification of IHC and OHC survival (from myosin7A, upper graphs) and pyknotic hair cell nuclei (from DAPI images, lower graphs) in control C, 250 μM neomycin-treated (N), phenoxybenzamine alone (C+PB) or neomycin and phenoxybenzamine treated (N+PB) explants. N numbers: Neo (11); Neo+PB (8); PB alone (9); control (10). *p < 0.05 (compared to control). Scale bar, 20 μm.

Given the requirement for drug pre-incubation, in test explants phenoxybenzamine (50, 100 or 200 μM) was applied for 1 h prior to the addition of neomycin in the presence of phenoxybenzamine. After 24 h explants were fixed, the same triple-labeling protocol was applied to all explants and image stacks collected and analyzed to quantify the number of surviving and pyknotic hair cells. At a concentration of 50 μM phenoxybenzamine did not offer significant protection against neomycin-induced ototoxicity (data not shown). In the presence of 100 μM phenoxybenzamine, however, the number of surviving OHC and IHC per 100 μm length of the cochlea was significantly higher, 27.9 ± 3.5 and 9.0 ± 1.0 respectively, compared to 17.6 ± 3.1 and 5.0 ± 1.4 with neomycin alone (*p* < 0.05). Using either the untreated control or phenoxybenzamine-treated alone explants to calculate the percent survival reveals that phenoxybenzamine increased OHC survival from 47% to 86% and IHC survival from 46% to 98% (Figure 6). Although phenoxybenzamine significantly reduced the number of neomycin-induced pyknotic OHC nuclei, we also observed a small but significant increase in pyknotic OHC and IHC nuclei when phenoxybenzamine was applied alone indicating the potential toxicity of the drug in the cochlea. The phenoxybenzamine-induced toxicity was more evident, particularly in OHCs at the highest concentration used (200 μM). In addition we observed that at 200 μM, rather than protecting hair cells against neomycin, phenoxybenzamine significantly enhanced the neomycin-induced toxicity in OHCs, whereas enhancement of toxicity was not observed in IHC (Supplementary Figure S1).

## Discussion

Phenoxybenzamine was one of a small number of compounds identified from two high-throughput drug screens for neomycin ototoxicity using zebrafish larvae (Ou et al., 2009; Vlasits et al., 2012). In zebrafish lateral line hair cells, the blockade of neomycin uptake into hair cells is thought to be the primary mode of action for the otoprotective effect of phenoxybenzamine (Ou et al., 2009). Here we used mammalian cochlear explant cultures from postnatal mice to evaluate the possible otoprotective effect of phenoxybenzamine. Testing compounds in the mammalian system is critical given the clear differences between the mammalian cochlea and the lateral line, not least because there are two distinctly different types of hair cell in the cochlea. We first characterized the effect of phenoxybenzamine on rapid FM1-43 uptake, which we use as mimic of neomycin entry since it permeates the mechanoelectrical transducer channels and has been shown to reduce the toxic effects of neomycin (Gale et al., 2001).

In zebrafish hair cells phenoxybenzamine blocks the uptake of both FM1-43 and gentamicin-Texas Red (Ou et al., 2009; Vlasits et al., 2012). We now show that phenoxybenzamine can block FM1-43 entry in both IHCs and OHCs in the mammalian cochlea. The IC50 for the blocking effect was quite similar between the two hair cell types (40–60 μM), however there was a ~4 fold difference in the Hill coefficient with the block in OHCs showing a steep dependence indicating cooperative binding, whereas in IHCs the coefficient was close to 1 suggesting non-cooperative binding of phenoxybenzamine. To better understand the mode of action, we compared pre-incubation of phenoxybenzamine with simple co-application and found that maximal inhibition was achieved by pre-incubation (Figure 2) as previously described in zebrafish (Vlasits et al., 2012). A 30 min pre-incubation was already maximal and no further block was observed when we extended the time of incubation to 60 min. Phenoxybenzamine is known to bind covalently to adrenergic receptors and serotonin receptors and in zebrafish the hair-cell survival effected by phenoxybenzamine persisted after phenoxybenzamine washout (Ou et al., 2009). In our live FM1-43 uptake assay, although the phenoxybenzamine effect remained for the first minute (at least in OHCs), the block was fully reversible after 60 min of washout. We observed differences in the effect of phenoxybenzamine on FM1-43 uptake in OHCs and IHCs. If the site of action of phenoxybenzamine in mammalian hair cells is within the mechanoelectrical transduction channel itself, this would suggest that there are significant differences in the structure of the channel between IHC and OHCs. Given the pre-incubation requirement, a simple explanation for this could that there is an intracellular binding site for phenoxybenzamine that is not present in IHCs. An alternative and perhaps more likely explanation is that there may be indirect effects of phenoxybenzamine on the mechanoelectrical transduction channels via regulatory molecules that are more prevalent in OHCs compared to IHCs.

We next examined whether the otoprotection seen in the lateral line with phenoxybenzamine is also observed in mammalian cochlear hair cells. The effects of AGs are both time and dose-dependent and we first set out to determine a neomycin-dose response curve for 24 h of exposure in our mouse cochlear explant model. We found a steep dose-dependence for neomycin-induced hair cell-death, particularly for OHCs (Hill coefficient of 4.7 compared to 1.9 in IHCs, Figure 5). The IC50 or 50% cell-death constant for neomycin was ~250 μM for both OHCs and IHCs and we used this concentration in subsequent assays in order to promote ~50% hair cell loss/survival, allowing either a protective effect or an enhancement of hair-cell death to be identified.

When used at a concentration of 100 μM phenoxybenzamine produced a significant protection of both OHC and IHCs, resulting in survival of both OHCs (86% survival) and IHCs (98% survival) relative to controls, i.e., phenoxybenzamine treatment alone. A small amount of toxicity was noted in the phenoxybenzamine-alone controls, based on the assessment of nuclear chromatin and pyknosis (Figures 1, 6). At a higher concentration, 200 μM phenoxybenzamine in combination with neomycin produced different effects on the two types of hair cell. The IHCs were still protected but there was an enhancement of toxicity in OHCs indicating an additive effect of phenoxybenzamine and neomycin. When phenoxybenzamine was applied alone at 200 μM for 24 h it resulted in a small amount of OHC death but did not affect IHCs. The stronger block of FM1-43 uptake in OHCs compared to IHCs that we have shown could explain the differential toxicity if we assume that activity of the MET channels is essential for cell survival. Another possible explanation for the phenoxybenzamine-induced toxicity observed in OHCs is that it results from the differential expression of known phenoxybenzamine targets. We checked cochlear gene expression on gEAR portal^1^ and found that both serotonin receptors and adrenergic receptors are differentially expressed by OHCs and IHCs (Liu et al., 2014) providing some support for this as an alternative hypothesis for the observed toxicity. Importantly, however, phenoxybenzamine protects both IHCs and OHCs from neomycin-induced toxicity and the simplest explanation is that this is due to a reduction in neomycin uptake via the MET channels, consistent with our FM1-43 uptake assay (a mimic for the neomycin uptake).

The lack of a significant effect of phenoxybenzamine on FM1-43 uptake in IHCs compared to OHCs, but the presence of a protective effect from neomycin suggests a greater complexity here. The primary pathway for the aminoglycoside entry into hair cells is now considered to be via the MET channels located at the top of the stereocilia, consistent with AGs acting as open-channel blockers (Kroese et al., 1989; Ricci, 2002; Marcotti et al., 2005) as described for FM1-43 (Gale et al., 2001). However, the contribution of other ion channels to the uptake of AGs into hair cells has been less well characterized. Channels such as the transient receptor potential (TRP) class members such as TRPC3, TRPV4, TRPA1 and PRPML3 which are expressed in the cochlea have not been ruled out as possible entry pathways (Castiglioni and García-Añoveros, 2007; Cuajungco et al., 2007; Asai et al., 2010; Stepanyan et al., 2011; Quick et al., 2012). AGs block a variety of ion channels such as large-conductance Ca^2+^-activated K^+^ channels (Nomura et al., 1990), N-type and P/Q type Ca^2+^ channels (Pichler et al., 1996), ryanodine receptors (Mead and Williams, 2002a,b), P2X receptors (Lin et al., 1993) and nicotinic acetylcholine receptors (Blanchet et al., 2000). Similar to its effects on the MET channels, FM1-43 is also known to block TRPC3/TRPC6 channels (Quick et al., 2012). Phenoxybenzamine could be affecting any of the above channels, however, investigating these different ion channels is outside of the remit of this current work. In these present experiments a somewhat unexpected result was the consistent difference we observed between IHC and OHCs. Differences in the nature of the MET channels between these two cell types have been described (Beurg et al., 2006, 2009) and although the our results were generated from a different type of assay, they support the possibility of there being alternative channel isoforms expressed in the two cell types.

In recent years high-throughput screening in non-mammalian models has proven to be very useful in identify new compounds for preventing hair-cell death. The data presented here indicate the importance of follow up studies in mammalian hair-cell models. Our study did identify consistency between results from zebrafish lateral line screens and the mammalian cochlea: from the mode of action of phenoxybenzamine on FM1-43 uptake to the requirement for pre-incubation to achieve maximal inhibition. The particular sensitivity of OHCs to phenoxybenzamine is, however, something that would tend to preclude its clinical use. Nonetheless, the differential effects of phenoxybenzamine on IHC and OHCs could turn out to be of significance in our understanding of the molecular mechanisms regulating MET channels.

## Author Contributions

All authors contributed to the initial conception and design of the work. PM undertook the majority of data analysis and drafted the manuscript. JEG contributed to data analysis and to the writing and revision of the manuscript. PAM collected data and undertook data analysis as part of an undergraduate project in Sussex and a Masters project at UCL. GR contributed to initial experimental design and manuscript revision.

## Conflict of Interest Statement

The authors declare that the research was conducted in the absence of any commercial or financial relationships that could be construed as a potential conflict of interest.

## Footnotes

^1^http://umgear.org/index.html

## Supplementary Material

Supplementary Figure 1 **Higher concentrations of phenoxybenzamine confer some protection against neomycin ototoxicity for IHCs but are toxic to OHCs. (A)** Quantification of hair cell survival in the presence of 200 μM phenoxybenzamine (PB) alone or in the presence of neomycin for both IHCs and OHCs. N numbers: Neo (11); control (4); Neo+PB 50 μM (4); 50 μM PB alone (4); Neo+PB 200 μM (5); 200 μM PB alone (4). **P* < 0.05 and ***P* < 0.005. **(B)** Representative confocal images showing F-actin (phalloidin), myosin 7a (Myo7a), for the hair cell soma and DAPI showing nuclear condensation and pyknosis in neomycin-treated examples.
