# Supplementary figures and images for: Protecting Mammalian Hair Cells from Aminoglycoside-Toxicity: Assessing Phenoxybenzamine’s Potential
Source: Front Cell Neurosci. 2017 Apr 18;11:94. doi: 10.3389/fncel.2017.00094 (PMC5408764; doi:10.3389/fncel.2017.00094)

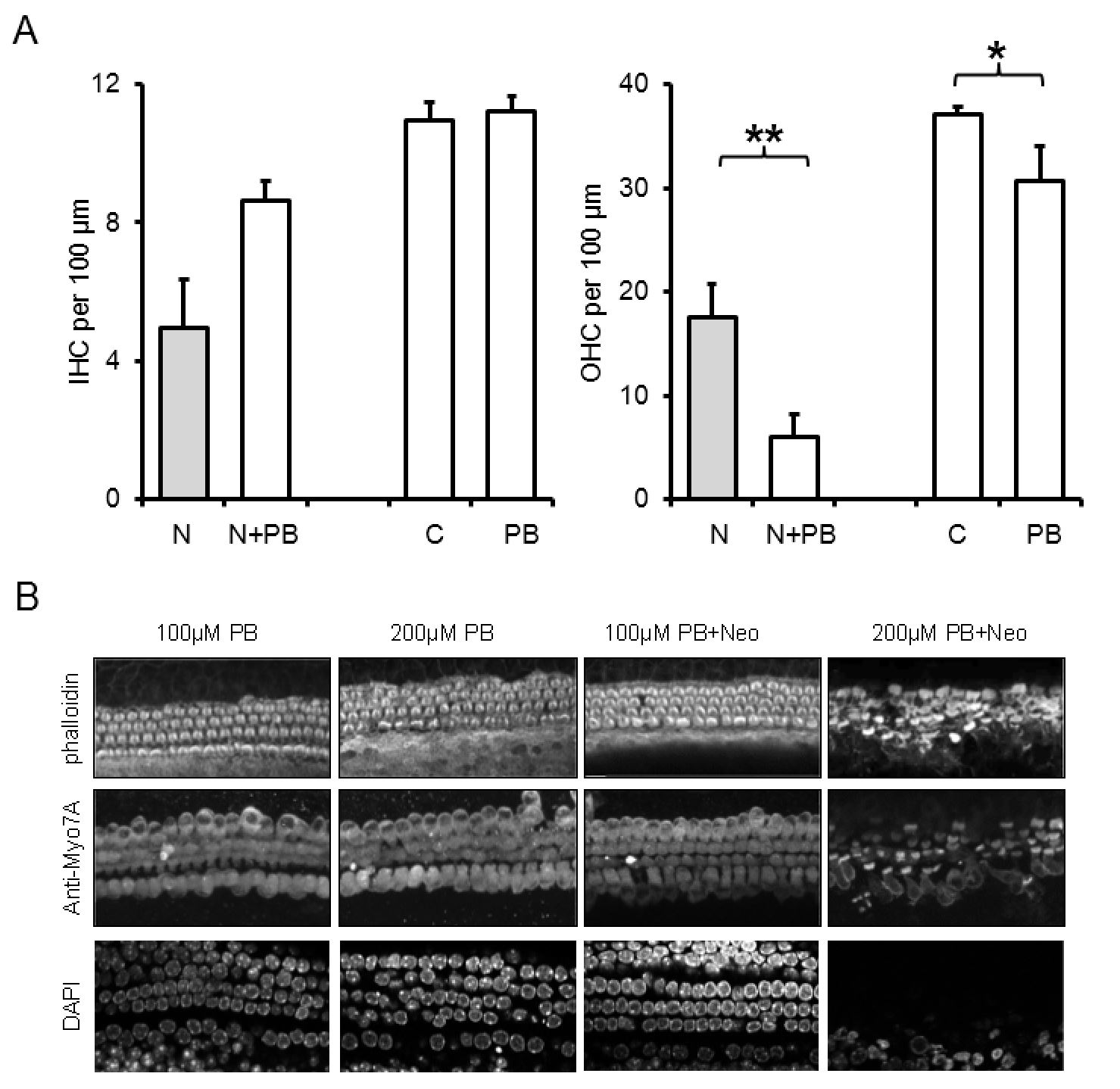

Supplement: Supplementary Figure 1 — Higher concentrations of phenoxybenzamine confer some protection against neomycin ototoxicity for IHCs but are toxic to OHCs. (A) Quantification of hair cell survival in the presence of 200 μM phenoxybenzamine (PB) alone or in the presence of neomycin for both IHCs and OHCs. N numbers: Neo (11); control (4); Neo+PB 50 μM (4); 50 μM PB alone (4); Neo+PB 200 μM (5); 200 μM PB alone (4). *P < 0.05 and **P < 0.005. (B) Representative confocal images showing F-actin (phalloidin), myosin 7a (Myo7a), for the hair cell soma and DAPI showing nuclear condensation and pyknosis in neomycin-treated examples. [file Image_1.jpeg]
